# Cardiovascular outcomes of metformin use in patients with type 2 diabetes and chronic obstructive pulmonary disease

**DOI:** 10.3389/fphar.2022.919881

**Published:** 2022-08-22

**Authors:** Fu-Shun Yen, James Cheng-Chung Wei, Lu-Ting Chiu, Chih-Cheng Hsu, Chii-Min Hwu

**Affiliations:** ^1^ Dr. Yen’s Clinic, Taoyuan, Taiwan; ^2^ Division of Allergy, Immunology and Rheumatology Chung Shan Medical University Hospital, Taichung, Taiwan; ^3^ Management Office for Health Data, China Medical University Hospital, Taichung, Taiwan; ^4^ College of Medicine, China Medical University, Taichung, Taiwan; ^5^ Department of Health Services Administration, China Medical University, Taichung, Taiwan; ^6^ Department of Family Medicine, Min-Sheng General Hospital, Taoyuan, Taiwan; ^7^ Institute of Population Health Sciences, National Health Research Institutes, Miaoli, Taiwan; ^8^ National Center for Geriatrics and Welfare Research, National Health Research Institutes, Yunlin County, Taiwan; ^9^ Section of Endocrinology and Metabolism, Department of Medicine, Taipei Veterans General Hospital, Taipei, Taiwan; ^10^ Faculty of Medicine, National Yang-Ming Chiao Tung University School of Medicine, Taipei, Taiwan

**Keywords:** coronary artery disease, heart failure, metformin, stroke, cardiovascular events

## Abstract

**Aim:** To know whether metformin use has different influence on cardiovascular risks in patients with type 2 diabetes mellitus (T2DM) and chronic obstructive pulmonary disease (COPD) as compared with metformin no-use.

**Methods:** This study employed a retrospective cohort study design. Using propensity score matching, we recruited 55 ,224 pairs of metformin users and nonusers from Taiwan’s National Health Insurance Research Database between 1 January 2000, and 31 December 2017. Cox proportional-hazards models with robust standard error estimates were used to compare the risks of cardiovascular outcomes.

**Results:** The mean study period of metformin users and nonusers was 11.04 (5.46) and 12.30 (4.85) years, respectively. Compared with the nonuse of metformin, the adjusted hazard ratios (95% CI) of metformin use for composited cardiovascular events, stroke, coronary artery disease, and heart failure were 0.51 (0.48–0.53), 0.62 (0.59–0.64), 0.48 (0.46–0.50), and 0.61 (0.57–0.65), respectively. The longer cumulative duration of metformin use had even lower adjusted hazard ratios compared with metformin nonuse.

**Conclusion:** In patients with coexisting T2DM and COPD, metformin use was associated with significantly lower risks of CVD; moreover, longer duration of metformin use was associated with a lower risk of CVD. A well-designed prospective study is required to verify the results.

## Introduction

Chronic obstructive pulmonary disease (COPD) is caused by smoking or air pollution and progressively limits the airflow. Furthermore, it can cause chronic inflammation of the respiratory tract and whole body and may exacerbate intermittently (Bhatt and Dransfield, 2013). Approximately 5%–10% of the population of the United States has COPD (Bhatt and Dransfield, 2013). Currently, COPD affects approximately 380 million individuals worldwide (Lopez et al., 2006). It is sixth among diseases in terms of global mortality and caused approximately 3.3 million deaths in 2019 (GBD 2019 Diseases and Injuries Collaborators, 2020). Type 2 diabetes mellitus (T2DM) is a hyperglycemic disease caused by insulin resistance and insufficient insulin secretion, which may be caused by obesity and reduced physical activity (International Diabetes Federation (IDF), 2019). The global prevalence rate of T2DM is approximately 9.3%. Currently, there are approximately 463 million patients with diabetes worldwide, and this figure is expected to increase to 578 million by 2030 (International Diabetes Federation (IDF), 2019). In 2019, it ranked eighth among diseases in terms of mortality (GBD 2019 Diseases and Injuries Collaborators, 2020).

T2DM, possibly through hyperglycemia-induced oxidative stress and inflammation, can cause pulmonary microvascular complications, diminish lung function, exacerbate COPD, and increase mortality (Gläser et al., 2015). Approximately 10% of people with T2DM also have COPD. Moreover, T2DM aggravates the progression and mortality risk of patients with COPD (Gläser et al., 2015). COPD, possibly through chronic inflammation and high-dose corticosteroid use, can lead to the development or deterioration of T2DM (Gläser et al., 2015). Therefore, COPD coexisting with T2DM is not uncommon, and it represents a relatively serious chronic disease that requires careful management (Gläser et al., 2015). Cardiovascular disease (CVD) shares many of the same causes with T2DM and COPD, such as old age, smoking, metabolic syndrome, and systemic inflammation (Bhatt and Dransfield, 2013). Therefore, CVD often occurs in patients with T2DM and COPD and is the leading cause of death among them (Bhatt and Dransfield, 2013; Low Wang et al., 2016). When using medications to treat patients with T2DM and COPD, it may be crucial to consider whether those medications have any effect on CVD risk.

Metformin has long been the world’s first-line medication for managing T2DM (American Diabetes Association, 2021). In the UK Prospective Diabetes Study (UKPDS), metformin use in overweight patients with T2DM was demonstrated to reduce the risk of myocardial infarction (UK Prospective Diabetes Study (UKPDS) Group, 1998). Its use in patients with COPD can cause lactic acidosis because they are prone to hypoxia; however, a later study confirmed that the incidence is very low (approximately 0.03 cases per 1000 person-years) (Bailey and Turner, 1996). Our previous study disclosed that metformin use in patients with COPD was associated with a reduced risk of mortality compared with metformin nonuse (Yen et al., 2018). Metformin exhibits antioxidant, anti-inflammatory, and possibly cardioprotective actions despite its glucose-lowering effect (Nesti and Natali, 2017). Therefore, we conducted this retrospective cohort study to investigate whether the influence of metformin use on CVD in patients with comorbid T2DM and COPD are different compared with metformin nonuse.

## Materials and methods

### Study population

Taiwan established its National Health Insurance (NHI) program in 1995. People are only required to pay a small premium, while their employers and the government pay the largest proportion (Cheng, 2003). By 2000, approximately 99% of Taiwan’s people had joined the NHI program. The NHI Research Database (NHIRD) contains data on the insured’s age, sex, place of residence, salary, medications, clinical procedures, and diagnosis according to the International Classification of Diseases, Ninth Revision, Clinical Modification (ICD-9-CM) and ICD-10-CM. The NHI Bureau regularly evaluates the diagnostic codes and medical management of clinical units to ensure correct diagnoses as well as the proper disposal of the NHI system. Additionally, to govern the usage of the NHIRD, Taiwan’s Ministry of Health and Welfare constructed the Health and Welfare Data Center (HWDC) in 2016 to standardize data management for all available health care data. All methods employed by this study were performed in accordance with the Declaration of Helsinki. The identifiable information of patients and caregivers was deidentified before release; therefore, the need for informed consent was waived. This study was approved by the Research Ethics Committee of China Medical University and Hospital (CMUH109-109-REC2-031).

### Study design

**FIGURE 1:**
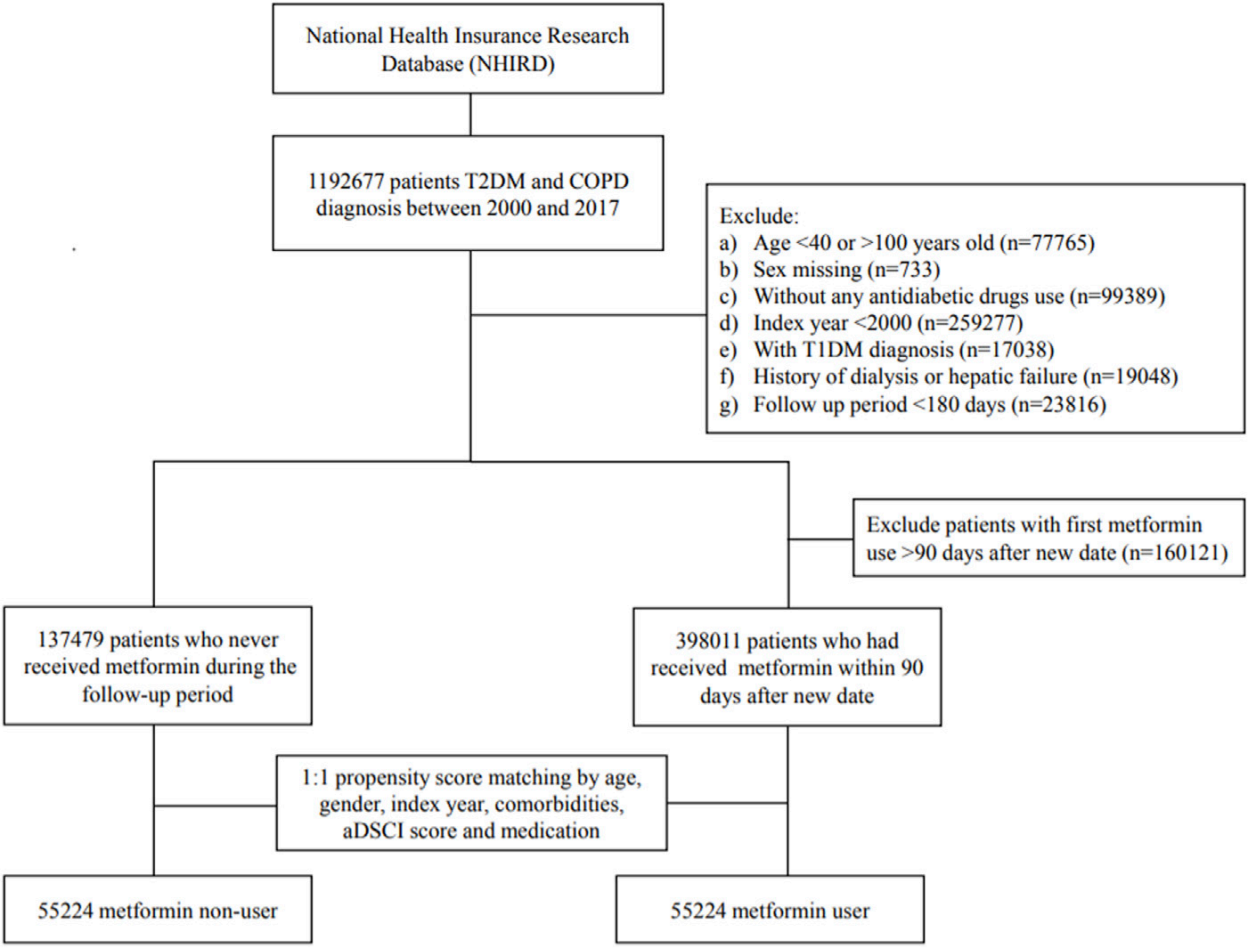
Flow diagram of the recruitment process.

We consecutively selected patients who had received the diagnoses of T2DM and COPD between 1 January 2000, and 31 December 2017; they were followed up to 31 December 2018. The diagnosis of T2DM and COPD was considered using the ICD-9-CM code or ICD-10-CM code (Supplementary Table S1) for at least two outpatient visits or one hospitalization record. Validation using ICD codes to define T2DM, COPD, and CVD has been performed by previous studies in Taiwan (Lin et al., 2005; Cheng et al., 2014; Lin et al., 2017; Su et al., 2019). We have further evaluated the severity of COPD. Mild-to-moderate COPD exacerbation was defined by the medication of antibiotics or systemic corticosteroids within 1 year, and which was managed in the outpatient setting. Severe COPD exacerbation was considered in the event of an emergency room (ER) visit or hospitalization within 1 year (Rodriguez-Roisin, 2000). Patients were excluded (Figure 1) if they (1) were < 40 or > 100 years; (2) did not use antidiabetic drugs; (3) had missing data of age or sex; (4) were diagnosed as having type 1 DM, hepatic failure, or were undergoing dialysis; (5) were followed up for less than 180 days after the index date to exclude latent diseases; or (6) had been diagnosed as having COPD or T2DM before January 1, 2000, to exclude prevalent diseases.

### Procedures

**FIGURE 2:**
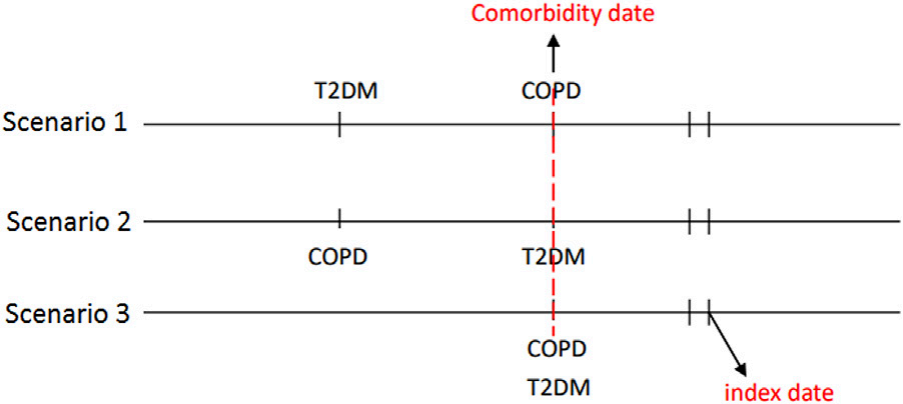
The concurrent diagnosis of T2DM and COPD was defined as the comorbidity date. The first date of metformin use after the comorbidity date was defined as the index date.

Comorbidity date was defined as the date of concurrent diagnosis of COPD and T2DM (Figure 2). Within 90 days following said date, patients who received metformin for at least 30 days were defined as metformin users, whereas those who never received metformin during the study period were defined as metformin nonusers. We defined the first date of metformin use after the comorbidity date as the index date. The index date of the control group was defined as the same period from the comorbidity date to the index date of the study group. Some related variables were assessed and matched between the metformin user and nonuser, including age, sex, comorbidity, Diabetes Complication Severity Index (DCSI) score and medication. Preexisting comorbidities including overweight; obesity; severe obesity; smoking status; the comorbidities of hypertension, dyslipidemia, chronic kidney disease (CKD), coronary artery disease (CAD), stroke, heart failure, peripheral arterial occlusive disease (PAOD), and atrial fibrillation diagnosed within 1 year before the index date; and medications use including respiratory drugs, antidiabetic drugs, antihypertensive drugs, statins, and aspirin during the study period. Glucagon-like peptide one receptor agonists were marketed in Taiwan since 2011, sodium-glucose cotransporter-2 inhibitors were marketed in Taiwan since 2015. Because the number of patients receiving these two antidiabetic medications was so small that they didn’t be contained in the baseline co-medication. To accurately reflect the characteristics of our patients with COPD and T2DM, we used the DCSI score (Young et al., 2008) to evaluate T2DM severity and the number of moderate or severe exacerbations of COPD to investigate the stability of COPD.

### Main outcomes

Hospitalized stroke, CAD, heart failure, and composite cardiovascular events were the main outcomes of this study. We calculated the events and incidence rates of composite cardiovascular events, stroke, CAD, and heart failure during the study period. The cumulative incidences of stroke, CAD, and heart failure were also compared between metformin users and nonusers.

### Statistical analyses

Propensity-score matching was used to match the related covariates between metformin users and nonusers (D’Agostino, 1998). The propensity score was estimated for each patient using nonparsimonious multivariable logistic regression, with metformin use as the dependent variable. We included 32 clinically relevant covariates as independent variables (Table 1). The nearest-neighbor algorithm was used to construct matched pairs, assuming the standardized mean difference of ≤ 0.05 to be a negligible difference between the case and comparison cohorts.

**TABLE 1:** Characteristics and medication use between metformin users and nonusers among patients with T2DM and COPD.

Characteristics	Before PSM					After PSM				
	Metformin nonusers		Metformin users		SMD	Metformin nonusers		Metformin users		SMD
	N = 1,37 ,479		N = 3,98 ,011			N = 55 ,224		N = 55 ,224		
	n	%	n	%		n	%	n	%	
Age, y										
<55	28** **,030	20.39	131** **642	33.07	0.28	14** **,252	29.91	12** **,833	26.93	0.04
55–65	35** **,742	26.00	132** **119	33.19	0.15	15** **,566	32.66	16** **,929	35.52	0.04
>65	73** **,707	53.61	134** **250	33.73	0.4	17** **,838	37.43	17** **,894	37.55	0.002
Mean ± SD	65.88 ± 12.02		60.46 ± 10.93		0.47	61.47 ± 11.02		61.78 ± 10.42		0.01
Sex										
Female	66** **,328	48.25	172** **661	43.38	0.1	24** **,970	52.40	24** **,628	51.68	0.009
Male	71** **,151	51.75	2,25 ,350	56.62	0.1	22** **,686	47.60	23** **,028	48.32	0.009
Comorbidity										
Obesity										
Overweight	147	0.11	556	0.14	0.009	70	0.15	64	0.13	0.003
Obesity	384	0.28	2048	0.51	0.03	225	0.47	200	0.42	0.007
Severe obesity	58	0.04	249	0.06	0.008	32	0.07	30	0.06	0.001
Smoking	1698	1.24	6491	1.63	0.03	8821	1.72	777	1.63	0.007
Chronic kidney disease	19** **,146	13.93	28** **,733	7.22	0.21	5107	10.72	5226	10.97	0.008
PAOD	4160	3.03	8033	2.02	0.06	1313	2.76	1341	2.81	0.003
Atrial fibrillation	2245	1.63	3780	0.95	0.06	517	1.08	511	1.07	0.001
Previous stroke	31** **,329	22.79	52** **,919	13.30	0.24	8319	17.46	8413	17.65	0.005
Previous CAD	45** **,317	32.96	94** **,474	23.74	0.21	14** **,770	30.99	14** **,751	30.95	0.009
Previous HF	6854	4.99	11** **,599	2.91	0.11	1719	3.61	1804	3.79	0.01
Exacerbation of COPD										
Mild	51** **,230	37.26	1,59 ,713	40.13	0.06	18** **,508	38.83	18** **,554	38.93	0.004
Moderate	74** **,310	54.05	2,20 ,326	55.36	0.03	26** **,758	56.15	26** **,654	55.93	0.002
Severe	11** **,939	8.68	17** **,972	4.52	0.17	2390	5.02	2448	5.14	0.006
DCSI score										
0	37** **,518	27.29	144** **371	36.27	0.19	15** **,851	33.26	15** **,865	33.29	0.001
1	17** **,762	12.92	55** **,773	14.01	0.03	7421	15.57	7253	15.22	0.009
2	82** **,199	59.79	197** **867	49.71	0.2	24** **,384	51.17	24** **,538	51.49	0.006
Medication										
Respiratory drugs										
β2 bronchodilator inhalants	59** **,154	43.03	149** **300	37.51	0.11	14** **,227	29.85	14** **,439	30.30	0.01
Anticholinergic inhalants	41** **,564	30.23	97** **,761	24.56	0.13	9007	18.90	9078	19.05	0.004
Corticosteroid inhalants	91** **,110	66.27	251** **303	63.14	0.07	30** **,674	64.37	30** **,701	64.42	0.001
Oral systemic corticosteroid	6954	5.07	16** **,320	4.10	0.05	1593	3.34	1561	3.28	0.003
Methylxanthine	42** **,011	30.56	1,00 ,381	25.22	0.11	9317	19.55	9456	19.84	0.007
Antidiabetic drugs										
Sulphonylurea	24** **,443	17.78	183** **854	46.19	0.95	17** **,821	32.27	17** **,881	32.38	0.002
TZD	16** **,153	11.75	1,00 ,740	25.31	0.73	12** **,928	23.41	13** **,170	23.85	0.02
DPP4 inhibitor	21** **,460	15.61	148** **219	37.24	1.07	11** **,128	21.09	11** **,641	21.08	0.003
AGI	21** **,776	15.84	1,25 ,181	31.45	0.69	16** **,225	20.15	11** **,381	20.61	0.01
Insulin	59** **,734	43.45	59** **,246	14.89	0.44	14** **,000	29.38	15** **,644	28.33	0.02
Antihypertensive drugs										
ACEI/ARB	40** **,428	29.41	1,03 ,747	26.07	0.07	14** **,837	31.13	14** **,754	30.96	0.003
β-blockers	46** **,534	33.85	109** **940	27.62	0.14	17** **,143	35.97	16** **,894	35.45	0.01
Calcium-channel blockers	31** **,461	22.88	74** **,171	18.64	0.1	11** **,325	23.76	11** **,234	23.57	0.004
Diuretics	32** **,546	23.67	82** **,521	20.73	0.07	12** **,426	26.07	12** **,270	25.75	0.007
Other drugs										
Statin	52** **,748	77.75	247** **547	85.11	0.19	37** **,402	78.48	37** **,545	78.78	0.007
Aspirin	70** **,016	50.93	228** **406	57.39	0.13	24** **,984	52.43	25** **,030	52.52	0.002

Data shown as n (%) or mean ± SD.

Abbreviations: ACEI, angiotensin-converting enzyme inhibitor; AGI, alpha-glucosidase inhibitors; ARB, angiotensin receptor blocker; CAD, coronary artery disease; COPD, chronic obstructive pulmonary disease; DCSI, diabetes complications severity index; DM, diabetes; DPP4 inhibitor, dipeptidyl peptidase-4, inhibitor; HF, heart failure; PSM, propensity-score matching; SMD, standardized mean difference; TZD, thiazolidinedione. A standardized mean difference of 0.05 or less indicates a negligible difference.

Crude and multivariable-adjusted Cox proportional-hazards models were utilized to compare outcomes between metformin users and nonusers. Results are presented as hazard ratios (HRs) and 95% confidence intervals (CIs) of metformin users compared with nonusers. To calculate the investigated CVD risks, we censored patients until the date of death, date of respective outcomes, or at the end of the follow-up on 31 December 2018, whichever occurred first. The Kaplan-Meier method and log-rank tests were used to compare the cumulative incidence of stroke, CAD, and heart failure during the follow-up period between metformin users and nonusers. We also assessed the cumulative duration of metformin use for the risk of stroke, CAD, and heart failure compared with metformin nonuse.

SAS (version 9.4; SAS Institute, Cary, NC, United States) was used for the statistical analyses; a 2-tailed *p* value < 0.05 was considered significant.

## Results

### Participants

From 1 January 2000, to 31 December 2017, 1 192 677 patients were diagnosed as having COPD and T2DM. After certain ineligible cases were excluded, we identified 398 ,011 metformin users and 137 ,479 nonusers during the study period. Figure 1 presents a flow diagram of the recruitment process.

Before propensity-score matching, we observed that the distribution of demographics, comorbidities and medications were significantly different between metformin users and nonusers (Table 1). After matching, 55 ,224 paired patients with COPD and t2DM were selected. In the matched cohorts, the mean (SD) age was 61.63 (10.72) years, and the mean T2DM duration was 2.02 (3.35) years. The mean study period of metformin users and nonusers was 11.04 (5.46) and 12.30 (4.85) years, respectively.

### Main outcomes

In the matched cohorts (Table 2), 9236 (16.72%) metformin users and 14 ,806 (26.81%) nonusers developed composite cardiovascular events during the follow-up period (incidence rate: 15.75 vs. 28.12 per 1000 person-years). In multivariable model, the metformin user had a significantly lower incidence of composite cardiovascular events compared with nonusers (aHR = 0.51, 95%CI = 0.48–0.53).

**TABLE 2:** Hazard ratios and 95% confidence intervals of hospitalization cardiovascular outcomes associated with metformin use.

Outcomes	Metformin nonusers (*n* = 55 224)			Metformin users(*n* = 55 224)			Crude HR (95% CI)	*p* value	Adjusted HR (95% CI)	*p* value
	Events	PY	IR	Events	PY	IR				
Composited cardiovascular outcomes	14** **,806	526** **554	28.12	9236	586 ,432	15.75	0.55 (0.54–0.56)	<0.0001	0.51 (0.48–0.53)	<0.0001
Stroke	6702	579 ,875	11.56	4614	605** **619	7.62	0.66 (0.63–0.68)	<0.0001	0.62 (0.59–0.64)	<0.0001
CAD	9728	559 ,808	17.38	5251	603 ,868	8.70	0.50 (0.48–0.52)	<0.0001	0.48 (0.46–0.50)	<0.0001
Heart failure	2220	612 ,224	3.63	1368	620 ,659	2.20	0.60 (0.57–0.64)	<0.0001	0.61 (0.57–0.65)	<0.0001

Abbreviations: CI, confidence interval; HR, hazard ratio; IR, Incidence rate = per 1000 person-years; PY, Person-years. aHR, adjusted for age, sex, comorbidities, DSCI, score, and medication listed in Table 1.

**FIGURE 3:**
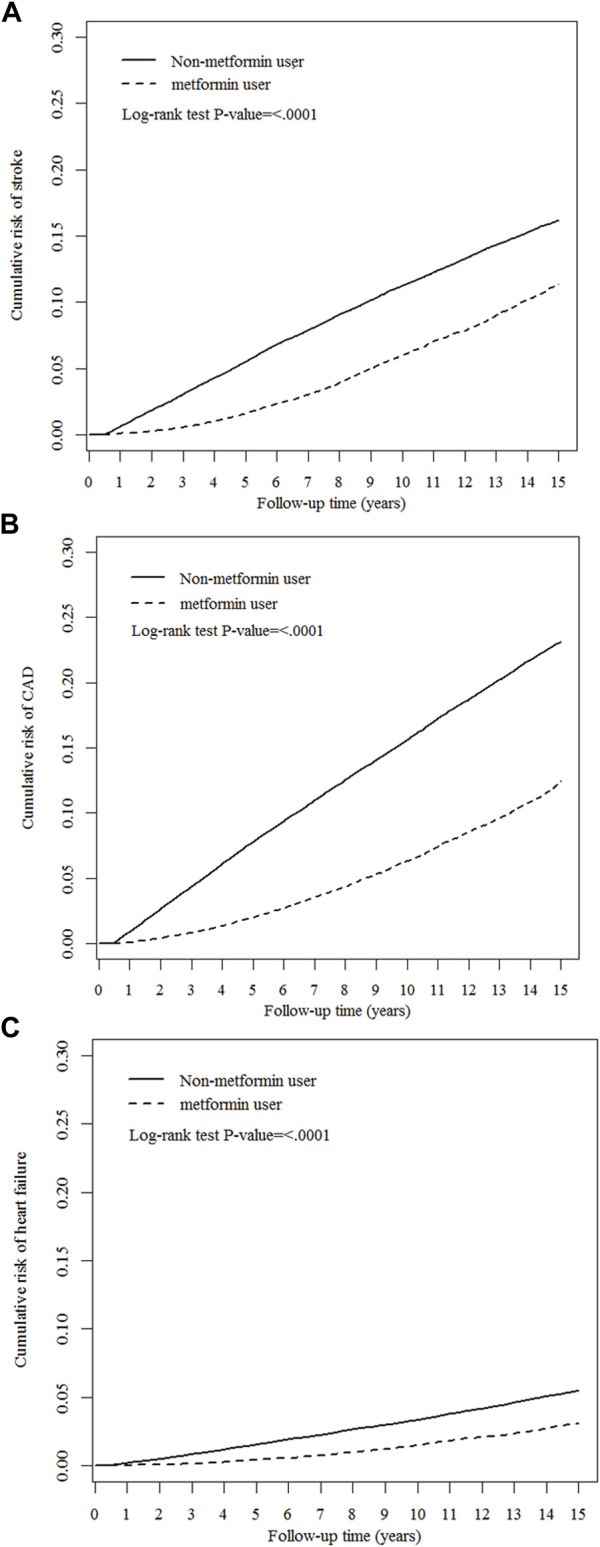
Cumulative risks of cardiovascular events between metformin users and nonusers. **(A)** stroke, **(B)** coronary artery disease (CAD), and **(C)** heart failure.

As presented in Table 2, compared with nonusers, metformin users were associated with significantly lower risks of hospitalized stroke (aHR 0.62, 95% CI 0.59–0.64), hospitalized CAD (aHR 0.48, 95% CI 0.46–0.50), and heart failure (aHR 0.61, 95% CI 0.57–0.65), respectively. The Kaplan−Meier analysis illustrated that the cumulative incidences of stroke, CAD, and heart failure were significantly lower in metformin users than in nonusers (Figure 3).

### Cumulative duration of metformin use

The association between the cumulative duration of metformin use and the risk of stroke, CAD and heart failure were further analyzed and presented in Table 3. We found that longer cumulative durations of metformin use were associated with lower risks of stroke, CAD, and heart failure; the *p* values for the trend of cumulative duration of metformin use in stroke, CAD and heart failure risk were less than 0.0001.

**TABLE 3:** Hazard ratios of cardiovascular outcomes associated with the cumulative duration of metformin use.

	Event	PY	IR	Crude HR (95% CI)	Adjusted HR (95% CI)
Cumulative duration of metformin use					
a) Stroke (*n* = 11 ,316)					
Nonuse	6702	579 ,875	11.56	1 (Reference)	1 (Reference)
<180 days	1962	186 ,538	10.52	0.91 (0.87–0.96)***	0.81 (0.77–0.86)***
180–360 days	761	97 ,788	7.78	0.67 (0.63–0.73)***	0.64 (0.60–0.69)***
361–720 days	846	129 ,633	6.53	0.56 (0.52–0.61)***	0.57 (0.53–0.61)***
>720 days	1045	191 659	5.45	0.46 (0.43–0.49)***	0.42 (0.40–0.45)***
*P* for trend				<0.0001	<0.0001
Cumulative duration of metformin use					
b) CAD (*n* = 14 ,979)					
Nonuse	9728	559 ,808	17.38	1 (Reference)	1 (Reference)
<180 days	2194	187 484	11.70	0.67 (0.64–0.71)***	0.64 (0.61–0.67)***
180–360 days	918	97 ,787	9.39	0.54 (0.51–0.58)***	0.53 (0.50–0.57)***
361–720 days	986	129 ,600	7.61	0.44 (0.41–0.46)***	0.43 (0.40–0.46)***
>720 days	1153	188 996	6.10	0.34 (0.32–0.36)***	0.31 (0.30–0.32)***
*P* for trend				<0.0001	<0.0001
Cumulative duration of metformin use					
c) Heart failure (*n* = 3588)					
Nonuse	2220	612 ,224	3.63	1 (Reference)	1 (Reference)
<180 days	516	184 674	2.79	0.78 (0.71–0.86)***	0.76 (0.69–0.84)***
180–360 days	216	98 ,218	2.20	0.61 (0.53–0.70)***	0.64 (0.56–0.74)***
361–720 days	276	133 018	2.07	0.57 (0.51–0.65)***	0.63 (0.56–0.71)***
>720 days	360	204 748	1.76	0.48 (0.43–0.53)***	0.44 (0.40–0.50)***
*P* for trend				<0.0001	<0.0001

^***^ *p* < 0.001.

Abbreviations: CI, confidence interval; HR, hazard ratio; IR, Incidence rate = per 1000 person-years; PY, Person-years. aHR, adjusted for age, sex, comorbidities, DSCI, score, and medication listed in Table 1.

## Discussion

This study demonstrated that metformin use in patients with T2DM and COPD was associated with significantly lower risks of composite cardiovascular events, CAD, stroke, and heart failure. In addition, a longer cumulative duration of metformin use was associated with an even lower risk of cardiovascular events.

Cardiovascular disease is the most critical complication of T2DM (Low Wang et al., 2016), and patients with T2DM for a longer duration are at higher risk of CVD (Sattar, 2013). Patients with COPD, possibly through the common pathogenic factors of aging, smoking, and chronic inflammation, are also prone to complications from CVD (Bhatt and Dransfield, 2013; Rabe et al., 2018). CVDs are the most common and lethal comorbidities in patients with T2DM and COPD (Bhatt and Dransfield, 2013; Rabe et al., 2018). Metformin has been reported to reduce the mortality risk in patients with COPD or chronic lower respiratory diseases (Yen et al., 2018; Ho et al., 2019; Mendy et al., 2019). The classic UKPDS also demonstrated that metformin use in overweight patients with T2DM had a significantly lower risk of mortality and myocardial infarction (MI), and this result persisted in the posttrial observational data (Holman et al., 2008). A randomized trial of insulin-treated patients with T2DM revealed that metformin use was associated with a lower risk of a composite cardiovascular endpoint compared with a placebo (Kooy et al., 2009). A meta-analysis of observational studies confirmed that metformin use was related to reduced MI risk compared with sulphonylureas use (Pladevall et al., 2016). Furthermore, metformin use in patients with T2DM was reported to be associated with a lower risk of hospitalization for heart failure in Taiwan (Tseng, 2019). However, a meta-analysis of randomized clinical trials of metformin use in patients with T2DM disclosed a neutral effect in reducing CVD risk (Griffin et al., 2017). This may be because of the small size of some studies as well as their short follow-up periods with low event rates. In brief, these studies have suggested that metformin may have beneficial cardiovascular effects, especially in certain populations such as overweight or insulin-treated patients (Nesti and Natali, 2017). Our study also disclosed that metformin use in patients with T2DM and COPD was associated with a prominently lower risk of CVD compared with metformin nonuse; furthermore, a longer duration of metformin use was associated with a lower risk of CVD.

Metformin, through inhibiting mitochondrial respiratory-chain complex 1, can activate the liver kinase B1 (LKB1)/adenosine 5′-monophosphate-activated protein kinase (AMPK) pathway, inhibit hepatic gluconeogenesis, reduce advanced glycation end products (AGEs), diminish oxidative stress and chronic systemic inflammation (Nesti and Natali, 2017). Metformin can also improve insulin resistance by activating tyrosine kinase, which can attenuate the sympathetic tone, decrease vasoconstriction, and reduce blood pressure (Nesti and Natali, 2017). Clinical and animal studies have demonstrated that metformin could reduce cardiac endothelial dysfunction (Sardu et al., 2019), attenuate cardiac ischemia reperfusion injury, and positively affect cardiac performance during acute heart stress (Bhatt and Dransfield, 2013; Nesti and Natali, 2017). Moreover, metformin can attenuate remodeling of the heart after cardiac injury as well as reduce cardiac hypertrophy (Bhatt and Dransfield, 2013). A clinical study disclosed that metformin can increase nitro-oxide production and vasodilatation as well as improve pulmonary hypertension (Agard et al., 2009). Metformin has also been reported to be associated with some improvement in lipoprotein levels (Bhatt and Dransfield, 2013; Nesti and Natali, 2017). Taken together, metformin may reduce CVD risk by reducing insulin resistance, systemic inflammation, endothelial dysfunction, cardiac injury and remodeling.

Our study has some strengths. First, this study started from 2000 to 2018, which could be sufficiently long, and the large number of patients to observe the effect of a drug or intervention on cardiovascular events. Second, the population was recruited from nationwide consecutive cases, and therefore, the selection bias may be minimal.

This study also has some limitations. First, the dataset lacked hemoglobin A1C, renal function, and other biochemical tests; moreover, it did not contain pulmonary function or cardioechogram results, precluding the precise calculation of diabetes and COPD severity scores. Instead, we used clinical records of the number of moderate and severe exacerbations to evaluate COPD severity, and the DCSI to evaluate the severity of T2DM. Second, the NHIRD is lack of complete information on dietary patterns, family history, and physical activity; accurate population data for alcohol consumption and smoking status are also difficult to obtain. The lack of such data may have influenced the results of metformin use and outcome assessment. Furthermore, we cannot completely rule out the possibility of confounding by indication; that is, metformin users may have a lower disease burden than nonusers who take insulin or other oral antidiabetic drugs. However, we performed propensity-score matching to optimally balance many relevant variables between the study and control groups to minimize bias from possible confounding factors. Third, we are never sure about the effective intake of metformin, because the blood metformin levels are not available. Fourth, this study was Chinese population–based research, and therefore, its result may not be applicable to other ethnicities. Finally, cohort studies inevitably face some unknown confounding factors, and thus, randomized controlled studies are required to verify our results.

COPD and T2DM are both common chronic diseases with a usually indolent evolution and systemic complications. CVDs are critical complications of T2DM and COPD, and usually underdiagnosed and under-treated in these patients (Holman et al., 2008). CVD can also worsen the prognosis of patients with T2DM and COPD. Our study demonstrated that metformin use was associated with a significantly lower risk of CVD compared with metformin nonuse. If metformin can truly reduce the risk of CVD in this high risk group of patients, it shall have the potential to modify the natural history of these diseases. A well-designed prospective study is warranted to confirm our results.

## Data Availability

Publicly available datasets were analyzed in this study. This data can be found here: Data of this study are available from the National Health Insurance Research Database (NHIRD) published by Taiwan National Health Insurance (NHI) Administration. The data utilized in this study cannot be made available in the paper, the supplemental files, or in a public repository due to the “Personal Information Protection Act” executed by Taiwan government starting from 2012. Requests for data can be sent as a formal proposal to the NHIRD Office (https://dep.mohw.gov.tw/DOS/cp-2516-3591-113.html) or by email to stsung@mohw.gov.tw.
